# Supplementary material for: Identification of photoperiod-induced specific miRNAs in the adrenal glands of Sunite sheep (Ovis aries)
Source: Front Vet Sci. 2022 Jul 22;9:888207. doi: 10.3389/fvets.2022.888207 (PMC9354845; doi:10.3389/fvets.2022.888207)
Supplement: Supplementary file 1 [file Data_Sheet_1.ZIP › Supplementary file/miRDeep2 file.zip!/miRDeep2 file/Know_Structure/SPLP42A1/oar-mir-485.pdf]

miRBase precursor : oar-mir-485
Total read count : 1709
oar-miR-485-5p read count : 630
oar-miR-485-3p read count : 1076
remaining reads : 3

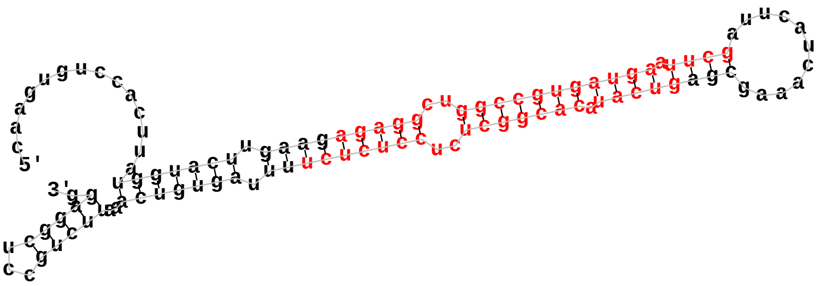

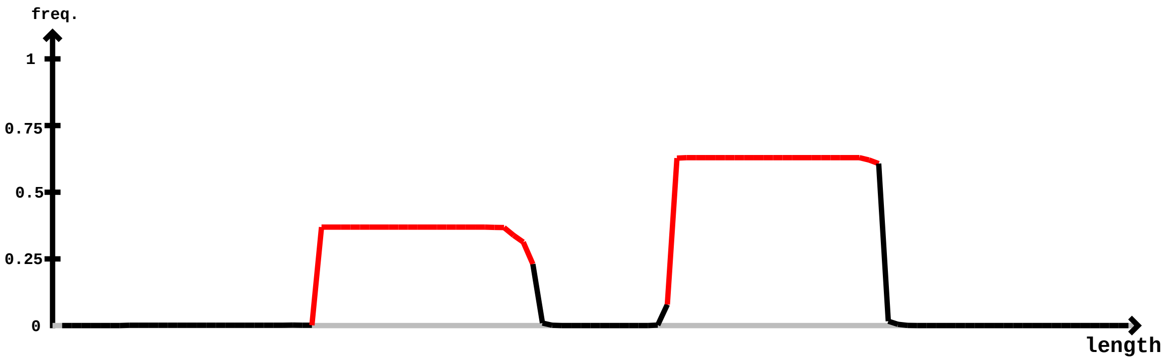

oar-miR-485-5p

oar-miR-485-3p

```
5'- caaguguccacuuaugguacuugaagagaggcuggccgugaugaauucgauucaucaaagcgagucauacacggcucuccucucuuuuagugucaaauucugccucggagg   -3'    exp
    ((((((.(((....)))))))))((((((((..((((((((((.((((..........))))))))).)))))))..)))))))).........(((((...))))).    reads      mm          sample
    ......uccacuuaugguacuuga.......................................................................................    1          0           seq
    ......uccacuuaugguacuugaag.....................................................................................    1          0           seq
    .......................aagagaggcuggccgugaugaauucgau............................................................    1          0           seq
    ..........................agaggcuggccgugauga...................................................................    2          0           seq
    ..........................agaggcuggccgugaugaa..................................................................    1          0           seq
    ..........................agaggcuggccgugaugaau.................................................................    50         0           seq
    ..........................agaggcuggccgugaugaauu................................................................    43         0           seq
    ..........................agaggcuggccgugaugaauuc...............................................................    141        0           seq
    ..........................agaggcuggccgugaugaauucg..............................................................    378        0           seq
    ..........................agaggcuggccgugaugaauucga.............................................................    14         0           seq
    ..........................agaggcuggccgugaugaauucgau............................................................    1          0           seq
    .............................................................gagucauacacggcucuccucu............................    1          0           seq
    .............................................................gagucauacacggcucuccucucu..........................    2          0           seq
    ..............................................................agucauacacggcucuccucu............................    2          0           seq
    ..............................................................agucauacacggcucuccucuc...........................    3          0           seq
    ..............................................................agucauacacggcucuccucucu..........................    125        0           seq
    ..............................................................agucauacacggcucuccucucuuu.......................    1          0           seq
    ..............................................................agucauacacggcucuccucucuuuu......................    1          0           seq
    ...............................................................gucauacacggcucuccucu............................    12         0           seq
    ...............................................................gucauacacggcucuccucuc...........................    20         0           seq
    ...............................................................gucauacacggcucuccucucu..........................    880        0           seq
    ...............................................................gucauacacggcucuccucucuu.........................    20         0           seq
    ...............................................................gucauacacggcucuccucucuuu........................    6          0           seq
    ................................................................ucauacacggcucuccucucu..........................    3          0           seq
```
